# A Clinical Case of COVID-19-Associated Pulmonary Aspergillosis (CAPA), Illustrating the Challenges in Diagnosis (Despite Overwhelming Mycological Evidence)

**DOI:** 10.3390/jof8010081

**Published:** 2022-01-14

**Authors:** P. Lewis White, Jan Springer, Matt P. Wise, Hermann Einsele, Claudia Löffler, Michelle Seif, Sabrina Prommersberger, Matthijs Backx, Jürgen Löffler

**Affiliations:** 1 Public Health Wales, Microbiology Cardiff, UHW, Cardiff CF14 4XW, UK; lewis.white@nphs.wales.nhs.uk (P.L.W.); Matthijs.Backx2@wales.nhs.uk (M.B.); 2 Medizinische Klinik und Poliklinik II, Universitätsklinikum Würzburg, 97080 Würzburg, Germany; Einsele_H@ukw.de (H.E.); Loeffler_C@ukw.de (C.L.); Seif_M@ukw.de (M.S.); Prommersbe_S@ukw.de (S.P.); Loeffler_j@ukw.de (J.L.); 3 Intensive Care Medicine, University Hospital of Wales, Cardiff CF14 4XW, UK; mattwise@doctors.org.uk

**Keywords:** *Aspergillus*, COVID-19, CAPA, diagnostics

## Abstract

The COVID-19 pandemic has resulted in large numbers of patients requiring critical care management. With the established association between severe respiratory virus infection and invasive pulmonary aspergillosis (7.6% for COVID-19-associated pulmonary aspergillosis (CAPA)), the pandemic places a significant number of patients at potential risk from secondary invasive fungal disease. We described a case of CAPA with substantial supporting mycological evidence, highlighting the need to employ strategic diagnostic algorithms and weighted definitions to improve the accuracy in diagnosing CAPA.

## 1. Introduction

COVID-19-associated pulmonary aspergillosis (CAPA) continues to be described in the literature, with case reports, case series, case/control studies, and prospective cohort studies leading to the development of consensus definitions to standardise the classification of CAPA. These international ECMM/ISHAM definitions recently appeared to provide clarity to the assorted CAPA classifications previously applied. Moreover, these also addressed questions of the accuracy of pre-consensus classifications, the varied incidences of CAPA, and the inconsistent mortality of some untreated CAPA cases given the scarcity of autopsy evidence [1,2]. Nevertheless, the ECMM/ISHAM definitions are not without limitations as they were defined pragmatically in response to a rapidly developing clinical situation, primarily in critically ill patients. Some diagnostics were, therefore, incorporated based on performance in other cohorts, with limited data available in the critical care COVID-19 patient. Some ‘probable CAPA’ classifications are achievable through the availability of a single positive mycological result. Nevertheless, applying these classifications across 37 studies describing CAPA generated an overall incidence of 7.6% for proven/probable/possible CAPA [3]. Their application to a recent multicentre evaluation of CAPA in the UK did not demonstrate a significant deleterious impact on mortality associated with a diagnosis of CAPA, even in the absence of antifungal therapy, [4] questioning the accuracy of the CAPA classification. It has been proposed that the classification of CAPA requires varying degrees of mycological evidence, dependent on the presence of supporting clinical/radiological evidence (e.g., chest radiology typical of invasive aspergillosis) [5,6].

We describe a case of CAPA in a critically ill patient with underlying myeloproliferative neoplasm and no significant immunosuppression. While CAPA cases are regularly being described, the level of mycological evidence available for this patient is unusual. The case demonstrates what is achievable when applying a strategic clinical algorithm incorporating multiple different diagnostics across a range of specimen types.

## 2. Case Presentation 

**Figure 1:**
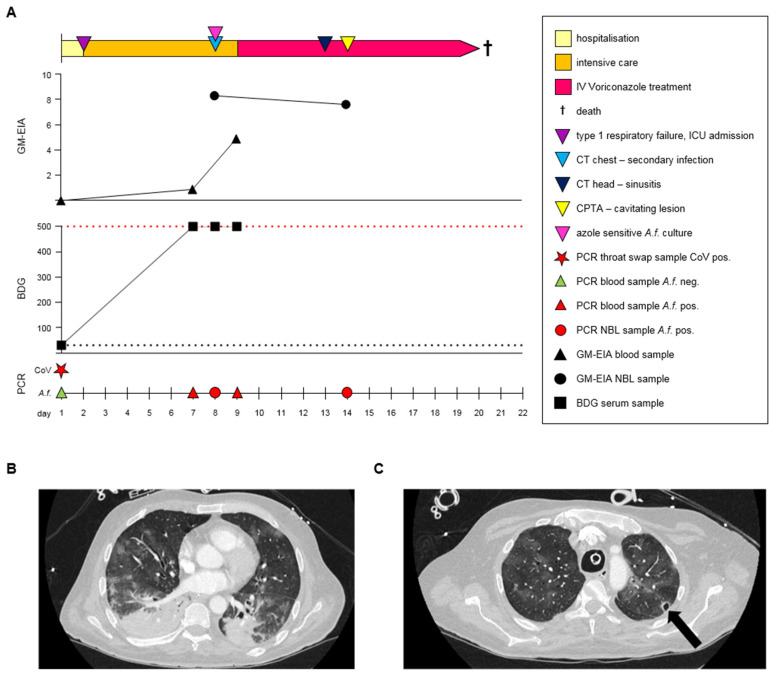
COVID-19-associated pulmonary aspergillosis (CAPA). (**A**) Clinical time course of CAPA in the case report patient. Key: BDG: (1-3)-β-d-Glucan, ICU: Intensive Care Unit, NBL: non-directed bronchial lavage fluid, IV: intravenous, GM-EIA: galactomannan enzyme immunoassay, CT: computed tomography scan, CTPA: CT pulmonary angiogram. (**B**) Extensive bilateral ground glass opacification and confluent consolidation. (**C**) CTPA showing ground glass opacification and a tiny new cavitary lesion in the left apex, indicated by the arrow.

In April 2020, a 58-year-old male patient with a history of Janus kinase (JAK)-2-positive essential thrombocythaemia, which had been treated with hydroxycarbamide, presented to the emergency assessment unit with severe respiratory distress requiring hospital admission (day 1) (Figure 1A).

He had been feeling unwell for several weeks with lethargy and loss of appetite being the predominant symptoms, which he related temporarily to a reduction in his hydroxycarbamide in response to falling haemoglobin (95 g/L) and low white blood cell counts (2.1 × 10^9^ cells/L: neutrophils 1.7 × 10^9^ cells/L, lymphocytes 0.3 × 10^9^ cells/L). On admission, the PaO_2_, PaCO_2,_ and SaO_2_ were 10.8 kPa, 3.5 kPa, and 95.8% respectively, on 2LO_2_ by nasal cannula. He was normotensive with a sinus tachycardia of 130/minute, a respiratory rate between 24–28/minute, and a high fever of 39.4 °C. A chest radiograph (CXR) on admission demonstrated bilateral airspace opacification. A throat swab was positive for SARS-CoV-2 by reverse-transcription polymerase chain reaction (RT-PCR), confirming a diagnosis of COVID-19 pneumonia. As the patient had been unwell for several weeks, a serum (1-3)-β-d-Glucan (BDG; Fungitell assay, Associates of Cape Cod) was sent on admission following discussion with microbiology colleagues and was negative (<31 pg/mL). Retrospective testing of this sample by *Aspergillus* PCR and galactomannan enzyme linked immune sorbent assay (GM-EIA; *Aspergillus* Ag kit, Bio-Rad) were also negative. In addition to evidence of SARS-CoV-2 pneumonia, the patient was in acute renal failure (urea 24.5 mmol/L (typical range [TP]: 2.5–7.8) and creatinine 217 µmol/L (TP: 58–110)) with a raised CRP (140 mg/L) and lymphopaenia (0.4 × 10^9^ cells/L) but a normal total white cell count of 5.7 × 10^9^/L (TP: 4–11) and was not neutropaenic (5.1 × 10^9^ cells/L). Over the next 24 h, the patient’s respiratory failure rapidly progressed, necessitating intubation, sedation, and mechanical ventilation. The patient required renal replacement therapy and vasopressors to support his blood pressure, in addition to being mechanically ventilated.

Initial blood cultures (day 1 through day 3) grew coagulase-negative staphylococcus, but were negative from there on, as were cultures from the central venous catheter (CVC), although the patient received multiple intravenous (IV) antibiotics throughout admission. Culture of the respiratory tract and *Legionella* antigen testing were negative. Throughout the admission to critical care, the patient was negative by PCR for the presence of *Pneumocystis jirovecii* and respiratory viruses other than COVID-19.

On day 7, serum BDG was strongly positive (>500 pg/mL, retrospective *Aspergillus* PCR and GM were also positive (I = 0.9)), CXR showed non-specific signs of progression of pneumonia, and the patient was lymphopaenic (0.2 × 10^9^ cells/L), but not neutropaenic (16.5 × 10^9^ cells/L). As per institutional protocol, a repeat BDG test was performed the following day (day 8), confirming the strong result and a non-directed bronchial lavage (NBL) fluid was positive by in-house *Aspergillus* real-time PCR and GM (I = 8.3) [7]. Computed tomography (CT) of the thorax and abdomen was performed on day 8 of his hospital stay. The imaging of the thorax demonstrated the progression of pneumonic changes with bilateral airspace shadowing and more extensive consolidation in the bases, which was worse on the right, raising concern of a secondary bacterial pneumonia (Figure 1B). There was evidence of splenic infarcts on the abdominal imaging with thickening and enhancement of the caecum and ascending colon. Dilated loops of small bowel with mesenteric fat stranding were observed. The differential diagnosis of the luminal findings included inflammation, infection, or ischaemia. In the presence of the splenic infarcts and possible gut ischaemia, therapeutic anticoagulation with heparin was initiated. On day 9 (day 8 of ICU admission), further strong BDG positivity in serum was supported by a positive *Aspergillus* real-time PCR and serum GM-EIA (I = 4.9), and the patient was commenced on voriconazole (IV, 300 mg twice daily) with a diagnosis of probable COVID-19-associated pulmonary aspergillosis (CAPA), classified in-line with international guidelines [1]. On day 14, the presence of *Aspergillus* in the respiratory tract (detected by GM and PCR assays, see Figure 1) persisted despite anti-fungal therapy and despite the isolation of azole-sensitive *Aspergillus fumigatus* from the NBL fluid obtained on day 8. A repeat NBL on day 14 yielded a positive *Aspergillus* real-time PCR and GM (I = 7.6). On day 13, a CT of the head showed evidence of sinusitis but no acute intracranial pathology, and on day 14, a CT-pulmonary angiogram highlighted a new small cavitary lesion at the apex of the left lung (Figure 1C). Despite ongoing clinical intervention, the patient remained in multi-organ failure and died on day 20.

## 3. Discussion 

We reported a case of probable CAPA with extensive mycological evidence and radiology typical of invasive aspergillosis. The patient had positive mycological evidence in the respiratory tract (*Aspergillus* PCR, GM-EIA, and *Aspergillus* culture of NBL) and circulation (*Aspergillus* PCR, GM-EIA, and BDG), which is unusual and reflects the strategic algorithm incorporated to monitor for invasive fungal disease in our critical care cohort. Given the environmental and ubiquitous nature of *Aspergillus* species, in particular *Aspergillus fumigatus*, one could argue that while more frequent testing enhances the opportunity for detecting less invasive disease presentations, it likely increases the detection of *Aspergillus* contamination or test positivity through chance. While this patient was positive across four different tests, applied to two different specimen types, sampling was not excessive. In total, three non-directed bronchial lavages were taken; one of the three was *A. fumigatus* culture positive and two had GM-EIA and *Aspergillus* PCR performed, and all were strongly positive. Four serum samples were taken; three were BDG positive (the only negative on the day of admission), and one serum sample had GM-EIA and *Aspergillus* PCR prospectively performed and was positive by both tests. Subsequently, over a two-week period, 10 of 13 fungal tests were positive, providing substantial evidence of CAPA.

The development of a cavity within the patient’s lung, a sign typically associated with invasive aspergillosis, provides evidence of invasion/disease progression and may explain the significant number of positive mycological tests encountered in this patient. Although CAPA testing was limited during the days immediately post ICU admission, it appears CAPA developed after one week’s stay, in-line with expected presentation timeframes (10 days, interquartile range 5–16 days) [8]. The cavity rapidly appeared one week later, demonstrating acute disease development, with the patient dying five days later. Treatment with voriconazole was administered as soon as a diagnosis was confirmed and the organism was fully sensitive to the antifungal treatment choice, but disease progressed. The patient did not receive dexamethasone or IL-6 inhibitors, having presented prior to available guidance from the RECOVERY trial. The patient did receive IV hydrocortisone, but it appears this was administered after the primary diagnosis of CAPA. The patient was not neutropaenic but was lymphopaenic prior to and during COVID-19 infection. Our knowledge of risk factors for developing CAPA is increasing, and the largest study to date associated older age, dexamethasone, IL-6 inhibitors, and prolonged mechanical ventilation with CAPA [9]. Clinically, it was felt that the patient died from multi-organ failure and ischaemic bowel, with CAPA, rather than directly from CAPA, and this highlights the difficulties in managing complex critical care patients where fungal infection can take advantage of an already deteriorating patient.

Despite the laboratory offering BDG, GM, and *Aspergillus* PCR testing at least six days a week during the peaks of the pandemic, delays in returning results are unavoidable due to sample transport, batch testing, and acknowledgement of the result on the ward. The use of lateral flow assays for *Aspergillus* can be used as point of care tests, and within the laboratory, can be used to test single samples requiring urgent testing (e.g., broncho-alveolar lavage fluid), enhancing turn-around time. Interestingly, both the blood and NBL fluid from this patient were positive by the IMMY Sona *Aspergillus* lateral flow assay when tested retrospectively.

Positive histology, usually obtained through autopsy, correctly remains the only route to attaining a diagnosis of proven CAPA, but the incidence of proven CAPA generally remains low (2%, 11/677 in a recent systematic review), although higher rates (20%) have been documented in certain centres [2,10]. The low rate of proven confirmation questions the accuracy of CAPA diagnosis which is often based on non-specific clinical and radiological signs, likely already present in a critically ill COVID-19 patient, combined with mycological evidence, which may be limited. It may also reflect the disease pathogenesis in patients without the classical host factors for developing invasive aspergillosis, leading to limited tissue invasion, with disease restricted to the airways. However, it should be remembered that a proven diagnosis based on positive histology or culture from a tissue biopsy is a highly specific, but not necessarily a sensitive result, and negativity is not sufficient to exclude disease. In our patient, autopsy was not performed (likely a reluctance of relatives to provide consent, but also very difficult to incorporate such procedures during the peak of a pandemic that is associated with significant mortality rates), but radiology was typical of that associated with invasive aspergillosis, and this was combined with significant mycological evidence, and even in the absence of positive histology, a diagnosis of CAPA appears robust. Over the course of the first COVID-19 wave, there were 10 cases of CAPA associated with this individual ICU, as defined in our original study [6]. Three additional cases presented with extensive positive mycology comparable to the case currently presented. There were no significant construction works in the immediate proximity of this ICU, and to date, no attempts have been made to genotype any *Aspergillus* cultures that were recovered from the respiratory tract of patients. The number of documented CAPA cases associated with this ICU likely reflects the uptake of extensive screening for CAPA by the individual unit. However, the first COVID-19 wave corresponded with a particularly dry spell of weather in Wales, and high levels of circulating, airborne *Aspergillus* spores were documented in the local containment level III laboratory.

Of the 19 cases of CAPA diagnosed in the original study, 94.7% had multiple positive mycological results, 57.9% had radiology typical of invasive aspergillosis, all but one with significant mycological evidence [6].

If the ECMM/ISHAM CAPA definitions are applied to the Welsh critical care cohort (*n* = 135) from the first COVID-19 wave, then 18 cases of CAPA (5 probable/13 possible) are classified [1,6]. While this is a similar rate to the rate defined using the local classifications, where 19 CAPA cases were diagnosed, overall concordance when defining CAPA from the 32 patients with positive mycology was only 72%. Overall mortality associated with CAPA defined using the local classification was 58%, being 47% and 100% in those receiving or not receiving appropriate antifungal therapy, respectively. Applying the ECMM/ISHAM definitions generated a similar overall mortality of 50%, but this was similar if the patient received appropriate antifungal therapy (46%) or not (60%). Of the four patients defined with CAPA by a single positive mycological result according to the ECMM/ISHAM classification, mortality was 50%, irrespective of antifungal treatment.

The use of weighted definitions requiring non-specific clinical/radiological evidence combined with multiple positive mycological results or radiology typical of invasive aspergillosis requiring at least a single positive mycological result may enhance the classification of CAPA in the absence of a proven diagnosis and should be considered when/if international definitions are reviewed.

## Data Availability

Not applicable.
